# Experiential Education Builds Student Self-Confidence in Delivering Medication Therapy Management

**DOI:** 10.3390/pharmacy5030039

**Published:** 2017-07-11

**Authors:** Wendy M. Parker, Kirsten M. Donato, Katie E. Cardone, Jennifer Cerulli

**Affiliations:** Albany College of Pharmacy and Health Sciences, 106 New Scotland Avenue, Albany, NY 12208, USA; Kirsten.donato@acphs.edu (K.M.D.); katie.cardone@acphs.edu (K.E.C.); cerullij@gmail.com (J.C.)

**Keywords:** medication therapy management, self-confidence, advanced pharmacy practice experience, experiential education, pharmacy education

## Abstract

To determine the impact of advanced pharmacy practice experiences (APPE) on student self-confidence related to medication therapy management (MTM), fourth-year pharmacy students were surveyed pre/post APPE to: identify exposure to MTM learning opportunities, assess knowledge of the MTM core components, and assess self-confidence performing MTM services. An anonymous electronic questionnaire administered pre/post APPE captured demographics, factors predicted to impact student self-confidence (Grade point average (GPA), work experience, exposure to MTM learning opportunities), MTM knowledge and self-confidence conducting MTM using a 5-point Likert scale (1 = Not at all Confident; 5 = Extremely Confident). Sixty-two students (26% response rate) responded to the pre-APPE questionnaire and *n* = 44 (18%) to the post-APPE. Over 90% demonstrated MTM knowledge and 68.2% completed MTM learning activities. APPE experiences significantly improved students’ overall self-confidence (pre-APPE = 3.27 (0.85 SD), post-APPE = 4.02 (0.88), *p* < 0.001). Students engaging in MTM learning opportunities had higher self-confidence post-APPE (4.20 (0.71)) vs. those not reporting MTM learning opportunities (3.64 (1.08), *p* = 0.05). Post-APPE, fewer students reported MTM was patient-centric or anticipated engaging in MTM post-graduation. APPE learning opportunities increased student self-confidence to provide MTM services. However, the reduction in anticipated engagement in MTM post-graduation and reduction in sensing the patient-centric nature of MTM practice, may reveal a gap between practice expectations and reality.

## 1. Introduction

In the United States, the Medicare Modernization Act of 2003 led to the creation of Medicare Part D prescription medication coverage along with the introduction of medication therapy management (MTM) as a required patient-centric service for high-risk beneficiaries with multiple prescription medications [1]. MTM seeks to optimize medication use outcomes through increased patient self-efficacy and medication adherence, while reducing adverse medication events [1]. Medicare-sponsored MTM services have evolved over the past decade to further define program deliverables and the vulnerable population it should serve. Medicare Part D MTM programs must include an annual offer to provide a person-to-person comprehensive medication review (CMR) of all medications and supplements followed by the provision of standardized written “take-away” materials for the patient (personal medication list and medication action plan) and targeted quarterly medication reviews. In addition to Medicare-sponsored MTM services, U.S. pharmacists provide similar services to non-Medicare beneficiaries and varied patient populations, which may or may not be covered by health insurance, prescription insurance or state-based Medicaid plans, and may include patient self-pay models. A vast range of services may be offered under the “MTM” umbrella term and implementation of the services are often location, payer, and provider specific [2]. Over the past decade, the pharmacy profession has generated similar but disparate definitions of MTM, patient-centered care and comprehensive medication management [3,4,5,6]. However, all involve pharmacist provision of a patient-centered review of medications with a goal of optimizing medication use outcomes using an integrated, team-based approach.

As demand for MTM services increases, colleges of pharmacy and the profession seek to educate students on the business, care delivery, and policy aspects of MTM prior to graduation to ensure preparedness for practice [7,8,9]. U.S. Doctor of Pharmacy accreditation standards indicate that advanced pharmacy practice experiences (APPEs) provide the capstone location in the curriculum for students to develop the needed self-confidence for independent and collaborative practice [8]. For the profession to ensure optimal patient medication use outcomes, graduates must not only have the knowledge and skills needed to deliver MTM, but also the confidence to use these abilities to advance the profession [10]. Self-confidence does not measure the knowledge and skills learned, but rather whether the individual believes he/she can use knowledge and skills to execute an action [11]. Self-confidence is one factor that determines a person’s decision to engage in an activity, including MTM, as a future practitioner [11,12,13].

At our institution, MTM is taught and practiced at various points throughout the didactic curriculum, with the American Pharmacists Association Delivering Medication Therapy Management Services (APhA-MTM) certificate program offered as an elective opportunity. There are several MTM-focused APPEs offered by both faculty and non-faculty preceptors, enabling about 15% of our students to engage in direct patient care MTM learning opportunities. The core required community pharmacy APPE also provides a venue for students to engage in MTM services and completion of a CMR is required during this rotation. However, much remained unclear about the extent to which our students were exposed to and delivering MTM during APPEs and if these learning opportunities increased their self-confidence to deliver specific core components of MTM service delivery. A survey developed to measure the impact of APPEs on student self-confidence did not address MTM specific activities [11]. Assessments that did address student perceived self-confidence to provide MTM services were either completed by students engaged in didactic MTM experiences or utilized composite scores to track self-efficacy in several domains across a curriculum [14,15].

An exploratory study was undertaken to determine the ability of our APPE experiences to provide MTM learning opportunities, develop student knowledge of MTM service provision and enhance student self-confidence to deliver MTM services. Specifically, the APPE experiences of fourth year (P4) student pharmacists were assessed in a pre/post APPE framework to: 1. identify student exposure to MTM learning opportunities; 2. assess the student pharmacists’ knowledge of the core components of MTM service delivery; and 3. assess student pharmacists’ self-confidence in their ability to perform MTM services. The goal was to identify how students’ didactic and APPE experiences affected their understanding and practical application of MTM, to guide future instructional design for faculty and experiential education opportunities related to MTM service delivery. In addition, we hope to use this formative research to finalize a systematic tool to evaluate MTM-focused APPEs for our institution.

## 2. Materials and Methods

### 2.1. Study Design and Participants

A survey-based evaluation was conducted at the completion of the P3 year and repeated at the end of the P4 year for students who graduated from our institution in 2015. All students from both our main and satellite campuses were invited to participate in the study. The study was approved by the Institutional Review Board at the Albany College of Pharmacy and Health Sciences (#13-007) prior to implementation and subjects were provided informed consent at the onset of the survey.

### 2.2. Survey Tool and Administration

The self-administered, electronic survey was constructed by the research team to address the 3 study objectives (Survey instrument Supplementary). Six closed-ended questions captured demographic data and factors perceived to influence student self-confidence (e.g., grade point average and prior work experience) based on previous literature [11]. To understand student pharmacists’ exposure to MTM learning opportunities, three questions assessed general awareness of MTM, how they first learned about MTM and if they completed the American Pharmacists Association’s Delivering Medication Therapy Management in the Community (APhA-MTM) certificate course. In the post-survey, students were asked to identify if they had participated in an MTM-focused APPE or engaged in MTM service activities during any APPE rotation. These questions were combined to capture any exposure to MTM services during APPEs. For each of these rotations, students were asked to evaluate the rotation’s learning opportunity using a 5-point, end-anchored Likert scale (1 = strongly disagree, 5 = strongly agree).

Student knowledge of MTM was assessed by two methods. First, an open-ended question asked students to provide their own definition of MTM. A primary coder created the code frame (KD) for these open-ended responses from established MTM definitions [1,3,4]. Two coders (WP and KD) reached consensus on the overall themes for the code frame which were then applied to the data from the pre- and post-APPE open-ended question. The second knowledge assessment was through a series of questions asking students if the listed eight features of MTM service delivery represented their understanding of MTM by checking the box next to it. These features were identified by two pharmacy practice faculty engaged in MTM service delivery (KC and JC) based upon published definitions of MTM [1,3,4,5,6]. These closed-ended questions were reviewed for percentage agreement with each question.

The final survey section asked students to self-assess, using a Likert scale, their overall self-confidence to provide MTM services to patients and their self-confidence engaging in 10 components of MTM service delivery (1 = strongly disagree, 5 = strongly agree; 1 = not at all confident, 5 = extremely confident). These represent components of the pharmacy practice faculty investigators’ (KC and JC) MTM-focused APPE syllabi (learning objectives and outcomes) developed from the CMS Medicare and pharmacy profession definitions of MTM, in conjunction with Doctor of Pharmacy accreditation standards and curricular outcomes [1,3,4,5,8,9].

Because of the unique nature of the questions and the students’ limited exposure to this method of data collection, a usability pilot study was conducted with 5 student pharmacists, in person, to verify the clarity and understandability of the survey questions, as well as to estimate a time requirement for the survey implementation. These 5 students were randomly chosen from a pool of P4 Class of 2014 student pharmacists (*n* = 20) who had completed a MTM-related rotation and who were available for an in-person interview during the study period. Each pilot participant was asked to complete the survey, and then a member of our team trained in interviewing techniques and group facilitation (WP) assessed the participants’ understanding of the instrument and ability to respond to the survey. Finding no major hurdles in the usability testing, several minor revisions were made to the survey and it was developed in an electronic, web-based program, REDCap (Research Electronic Data Capture) (Vanderbilt University, Nashville, TN, USA), for secure, anonymous data collection.

Students were emailed a link via their college email addresses to the anonymous survey at the beginning of their APPE year (April/May 2014, pre-APPE) and at the end of the APPE year (April/May 2015, post-APPE). Two reminder e-mails were sent during both data collection periods and surveys were held open for 3–4 weeks each time. Survey respondents were not individually matched pre- and post-APPE year, as surveys were not linked to an individual, and no names, dates of birth, student IDs or other identifiable information were collected to ensure anonymity. Students could provide their names and email addresses at the conclusion of the survey (stored separately from survey data) to be entered into a raffle for one of five $25 gift cards as an incentive for participation.

### 2.3. Data Analysis

Results were tabulated for the pre- and post-assessment separately and, when appropriate, a t-test for a test of significance was computed to evaluate the differences between the assessments or between relevant sub-groups within an assessment for continuous data. Chi-square tests were used to evaluate difference in proportions between the pre- and post-test responses. Analysis was completed via SAS^®^ (9.4). All tests were evaluated at a 5% level of significance.

## 3. Results

### 3.1. Students’ Demographic Characteristics

There were 238 graduates in 2015, with 180 on the main campus and 58 at the satellite campus. Responses were received from 62 students, representing a 26% response rate in the pre-test; 44 responses were received in the post-test (18% response rate). Respondent demographics are provided in Table 1. The respondents were mostly male and largely represented the main campus of the institution in proportions similar to the overall population of the college (overall male students = 56%; satellite campus students = 17% of 2015 graduating class). The majority of respondents reported a professional GPA of 3.0 or higher on a 4.0 scale indicating overall above average grades in all their coursework. Of the respondents, more than 75% worked at a pharmacy either during college breaks, including winter and summer, or during the academic year. There were no significant differences in post-graduation career plans among respondents from the pre- to post-assessment periods. Slightly fewer students (23% to 16%) planned to pursue residency training, while more chose to work at a community chain in the post-assessment (32% to 52%).

### 3.2. Student Exposure to MTM Learning Opportunities

Nearly all respondents indicated they were aware of MTM and most learned about it in classes or from faculty (81% and 74%, respectively, Table 2). About half of students in the pre-test (48%) and post-test (50%) had completed the APhA MTM certificate program. More than half of students (68%) reported engaging in some MTM activities during their APPE experiences (Table 2). Students rated these MTM-related APPE experiences highly, between 4.4–4.6 out of 5, on a Likert scale with 5 indicating strong agreement with positive statements about the rotations.

### 3.3. Student Knowledge of Core Components of MTM

Student pharmacists defined ‘MTM’ in two ways to demonstrate their knowledge of the topic (Table 3), via open- and closed-ended survey responses. For the open-ended definition of MTM, data coding revealed three broad categories: assessing medication-related problems (MRPs), performing a CMR and communication with other health care professionals. About 70% of students indicated that MTM was defined through assessing MRPs in both pre- and post-test. Approximately 11% (11.6% and 10.8%; pre-test and post-test respectively) of respondents defined MTM through performing a CMR and one fifth (18.0% pre-test and 18.7% post-test) defined MTM as dealing with communication to primary care providers. These numbers were relatively consistent in the pre- and post-assessments. For the closed-end responses to the eight statements regarding features of MTM service delivery, there were similar patterns of agreement from pre- to post-test including that ‘MTM can be individualized for each patient’ and ‘MTM is a comprehensive approach to improving medication use’. This was true with one notable and significant exception; in the pre-test, almost 94% of respondents believed that MTM is patient-centric, while in the post-test, only 80% indicated agreement with that definition (*p* = 0.03). Although not statistically significant, there was a trend toward reduction in agreement with the statement that MTM “allows for an interactive person-to-person, patient and provider conversation” (pre-APPE = 93.6%, post-APPE = 81.8, *p* = 0.06).

### 3.4. Student Self-Confidence to Perform MTM

Students were asked to provide a self-assessment of their overall confidence to engage in MTM and the ten specific components of MTM (Table 4). Students reported increased overall self-confidence in providing MTM following their APPEs (3.27 to 4.02 mean rating; *p* < 0.0001) demonstrating the desired impact of APPE learning experiences. There were significant increases in self-confidence of student pharmacists’ abilities, assessed via the Likert scale noted earlier, to prepare for and conduct a CMR and to prepare patient deliverables (i.e., personal medication list, medication action plan), to communicate with the patient and providers regarding MRPs, to offer evidence-based, individualized recommendations, and to discuss MRPs verbally and in writing. Before respondents completed their APPEs, around 90% of people indicated that they would provide MTM in the future, but only 77% of respondents indicated they expected to practice MTM in the future in the post-test assessment (*p* = 0.07).

To evaluate what experiences might be helpful in enhancing MTM skills, subgroup analyses were conducted based on factors related to increased self-confidence in MTM abilities [11]. Students who work during pharmacy school, had a GPA > 3.5 (academic high achievers), or who completed the APhA MTM certificate course all started off more confident in their abilities to complete MTM and its associated core components (e.g., constructing a personal medication list, identifying MRPs, etc.) when compared to the overall population, but the pattern of growth following APPE experiences was similar to the overall population and did not result in significantly more self-confidence in the post-test. However, students who reported engaging in MTM activities during APPEs did demonstrate significantly more confidence in provision of MTM services (Table 5). (Additional sub-group analyses available upon request).

## 4. Discussion

Ensuring pharmacy graduates are adequately prepared to provide MTM services is important to meet the needs of the patient population. Popovich eloquently stated that pharmacy students need exposure to new information (knowledge and skills) and then, need to experience the application of that information to cultivate the intellectual confidence required to enable action [10]. The concept that confidence is required for action holds true for practitioners as pharmacists’ self-confidence to provide MTM services was identified as a significant factor in their likelihood to provide MTM in the future [12].

This present study demonstrated that our students’ didactic learning opportunities provided a good foundation to engage in MTM service delivery upon entering APPE with baseline knowledge of the process. Most students (68%) were engaged in MTM-related activities during APPEs, which significantly increased their self-confidence to practice MTM above those who did not report receiving MTM learning opportunities during APPEs. This demonstrates the activities are developing the confidence desired, however, it indicates a potential need to increase experiential exposure to MTM services above 68%, as all our students should garner patient-centered care experiences. Or, is it possible that there were additional students who participated in delivery of MTM services during APPEs, but who did not indicate such during the self-reported exposure? For example, students in ambulatory settings who were in fact conducting CMRs with patients and collaborating with providers at the site to optimize medication therapy, may not have defined the activity as MTM, perhaps because they were not billing for the service as “MTM” using the web-based platforms typically used to provide the services, which were discussed in the classroom and/or used in their work place. Disparate definitions of what activities constitute MTM may have affected student response to individual questions or completing the survey overall. In the US, the profession itself struggles with defining and identifying the services or activities which constitute MTM and how to define and represent pharmacist patient care activities in the electronic medical record [16]. We identified opportunities to build more consistency in how MTM is explained and defined across our curriculum and for APPE faculty and preceptors to assist in realigning student pharmacist expectations with real-world exposure. Providing students with a definition of MTM that encompasses the variations seen in practice and exposing a greater cohort of students to MTM training (i.e., the APhA-MTM certificate) could begin to build a more cohesive understanding of MTM services among our students [7].

Several efforts were made to reduce the possibility of response bias through a broad-based email invite asking for feedback overall on APPEs, by making the surveys anonymous at each point and completing the interviews online in an environment less likely to encourage social desirability. Additionally, the invite came from a member of the research team not within the school/program of pharmacy on campus, so there is limited reason to believe students would be trying to ‘say the right thing’ given they were unlikely to know that faculty member personally. Researchers attempted to achieve a good response rate by using the designated college email system (to avoid the email being marked trash or spam) and two email reminders. The low response rate could have been impacted by the survey coming from a faculty member who students may not have been familiar with. An additional factor impacting response could have been the first email for distribution of the survey was sent to students in the final week of the didactic curriculum when students find themselves busy with final exam preparation.

Following APPEs, student confidence to engage in MTM improved consistent with the desired accreditation standards and practice expectations, demonstrating the impact of the APPEs [8,10,11,15]. However, we wanted to explore further which factors could influence confidence to provide MTM services (e.g., GPA, work experience, the APhA-MTM certificate elective course, MTM APPE experience). Wongwiwatthananukit et al. suggested that student GPA was a predictor of overall self-confidence during APPEs, which is a factor one would expect to carryover to MTM-specific practice [11]. In a 2013 assessment of student self-confidence in MTM-related activities, investigators hypothesized student GPA or engagement in pharmacy work outside the curriculum could impact student self-confidence, however their data did not include these factors [14]. Most students responding to our survey obtained a GPA > 3.0 limiting the ability to engage in a detailed analysis of the impact of GPA on self-confidence. Students engaging in the APhA-MTM certificate course or who had worked in a pharmacy, had more confidence at the onset of the APPEs, but the APPE experiences brought those without those previous experiences to the same level of confidence. Students engaging in MTM specific activities during APPEs achieved a significant increase in confidence, consistent with the expectation that experiences in the application of information would increase confidence. Students who gained MTM experience not only increased confidence, but also were more likely to report they plan to practice MTM in the future, which is consistent with previous literature linking APPE experiences to increased confidence, and higher confidence to increased likelihood to engage in the future [12,15]. Although not a validation, garnering results consistent with expectations and previous literature demonstrate the utility of the survey as a method for our institution to obtain assessment data to ensure MTM-confident graduates. Because surveys were anonymous, a matched pre- to post-test could not be completed to evaluate how these factors could impact one’s individual growth. Additional work utilizing a matched pre/post design could evaluate in more detail how each factor (GPA, work experience, APhA-MTM certificate course, APPE experiences), or other yet unknown predictors, alter the self-confidence and interest in future MTM delivery for student pharmacists.

Two specific findings from this study leave us with more questions. Why did students indicate they are less likely to provide MTM after graduation? Why did fewer students indicate that MTM is patient-centric in the post survey? While students grew more self-confident in their abilities to deliver the core components of MTM service delivery, they ended the APPE year with fewer indicating likelihood to practice MTM post-graduation, even among those who gained MTM confidence and experience (Table 5). This contrasts with previous literature where higher pharmacist confidence increased the likelihood of engaging in MTM services [12]. Most indicated they would likely work in community pharmacy, where the opportunity to provide MTM-type interventions in the US continues to grow. However, in our specific region, some pharmacists do not regularly engage in comprehensive MTM services, while instead completing only a portion of MTM services (e.g., counseling or medication review) and may not see what they do as comprehensive MTM services. Additionally, there remain few compensation mechanisms for MTM services in certain settings (e.g., ambulatory care settings) or for non-Medicare Part D services, resulting in fewer opportunities for them to carry out MTM-type services. Perhaps students responded they were not likely to provide MTM after graduation not due to a lack of confidence in their ability or lack of exposure to MTM, but rather, a lack of confidence in obtaining a position where pharmacist responsibilities include MTM service provision. Students focus and plans may have changed over their final year of training—separate from MTM experiences—that may have led them in a different career direction. Responses could also be an indication of reduced interest in MTM. In any case further investigation is warranted to better understand these results.

The reduction in the perception that MTM is interactive and patient-centric may reflect how MTM is practiced by pharmacists and experienced by the students in the real-world setting. Many regional pharmacists and students engage in targeted interventions for specific medication-related problems or telephonic adherence/refill reminder programs considered part of MTM service provision. While these activities constitute a component of MTM service delivery, they do not represent the highly interactive pharmacist-patient-prescriber communications typical of a CMR [3,4,5,6]. Students’ MTM learning experiences may not be quite 'living-up' to their expectations for patient or provider interactions.

## 5. Conclusions

Students gained self-confidence to engage in the core components of MTM following the opportunity to engage in MTM related activities during APPEs. Therefore, colleges should continue to verify the availability of high level APPE MTM learning opportunities for all students. To ensure systematic integration of MTM across the curriculum and set appropriate practice expectations, we recommend that pharmacy curriculums continue to emphasize a consensus definition of MTM as disparate definitions of what constitutes “MTM” may cloud expectations.

## Figures and Tables

**Table 1:** Student Demographics.

Characteristic	Pre-APPE *N* = 62 *n* (%)	Post-APPE *N* = 44 *n* (%)	*p* Value
**Age**			0.03
21–23	27 (43.5)	9 (20.5)	
24–26	22 (35.5)	18 (40.9)	
27 and older	13 (21.0)	17 (38.6)	
**Sex**			0.55
Male	33 (53.2)	26 (59.1)	
Female	29 (46.8)	18 (40.9)	
**Campus**			0.43
Main	53 (85.5)	35 (79.5)	
Satellite	9 (14.5)	9 (20.5)	
**Professional GPA**			0.11
3.5 or greater	14 (22.6)	14 (31.8)	
3.0–3.49	29 (46.8)	25 (56.8)	
2.5–2.99	17 (27.4)	5 (11.4)	
2.0–2.49	2 (3.2)	0 (0)	
**Work in Pharmacy**			
during school	32 (51.6)	23 (52.3)	0.95
during break	47 (75.8)	33 (75.0)	0.92
**Post-Graduation Employment Plans**			0.58
Community chain	20 (32.3)	23 (52.3)	
Community, independent	3 (4.8)	3 (6.8)	
Community, mass merchandiser	2 (3.2)	4 (9.1)	
Grocery store	5 (8.1)	1 (2.3)	
Residency	14 (22.6)	7 (15.9)	
Fellowship	1 (1.6)	1 (2.3)	
Graduate school	8 (12.9)	1 (2.3)	
Academia	3 (4.8)	1 (2.3)	
Industry	2 (3.2)	1 (2.3)	
Managed care	2 (3.2)	1 (2.3)	
Other	2 (3.2)	1 (2.3)	

**Table 2:** Student Exposure to Medication Therapy Management (MTM) Learning Opportunities.

	Pre-APPE *N* = 62	Post-APPE *N* = 44	*p* Value
**Are You Aware of What MTM Services or Programs Are? *n* (%) Yes**	58 (93.5)	42 (95.5)	0.52
**How Did You First Learn of MTM? [Open Ended Coding, *n* (%) within Code for Those Responding to Question]**			
School/Classes/Faculty	50 (80.6)	32 (74.4)	
Experiential Education	2 (3.2)	1 (2.3)	
Work/employment	7 (11.3)	10 (23.2)	
Unaware of MTM	3 (4.8)	0	
**Exposure to MTM Learning Opportunities**			
Did you complete the APhA MTM certificate training elective? *n* (%) yes	30 (48.4)	22 (50.0)	0.87
Did you engage in MTM during APPE? *n* (%) yes		30 (68.2)	
If yes, in which type of APPE did you engage in MTM? *n* (%) yes *			
Community Pharmacy		15 (55.6)	
Inpatient Clinical		0	
Institutional/Hospital		0	
Ambulatory Care		3 (11.1)	
Elective		9 (33.3)	
For APPE that Incorporated MTM, Indicate Agreement with the Statement Below (1 = Strongly Disagree; 5 = Strongly Agree) Mean (SD) *			
This rotation met my educational needs		4.56 (0.64)	
The rotation added to my development as a pharmacist		4.52 (0.64)	
This rotation provided me with useful skills that should help me obtain a job		4.44 (0.70)	

* 3 missing data, *n* = 27. MTM = Medication Therapy Management; APhA = American Pharmacists Association; APPE = Advanced Pharmacy Practice Experience.

**Table 3:** Student Knowledge of MTM.

	Pre-APPE ^+^	Post-APPE ^++^	
Definitions (Open Coding)	62 respondents 189 codes	44 respondents 139 codes	
Respondent provided any knowledge of MTM services/programs in comments [SUMMARY CODE, *n* (%) codes]	61 (98.4)	43 (97.7)	
Assessing MRPs, *n* (%) codes	133 (70.4)	98 (70.5)	
Assessment of patient health	15 (11.3)	11 (11.2)	
Review, identify, resolve, and prevent MRPs	41 (30.8)	25 (25.5)	
Administering, selecting, initiating, and modifying medication therapy	24 (18.1)	15 (15.3)	
Safety and effectiveness via patient response to therapy	24 (18.1)	22 (22.4)	
Information, support services, resources for adherence and how to optimize	29 (21.8)	25 (25.5)	
Perform a CMR, *n* (%) codes	22 (11.6)	15 (10.8)	
Perform a CMR to identify, resolve, and prevent MRPs	6 (27.3)	2 (13.3)	
Verbal education and training for patient understanding	16 (72.7)	13 (86.7)	
Communicate to other HCP, *n* (%) codes	34 (18.0)	26 (18.7)	
Documentation and communication to HCP	9 (26.5)	8 (30.8)	
Coordinate MTM with broader healthcare services (reduce costs, better health outcomes)	25 (73.5)	18 (69.2)	
No Answer	1 (1.6)	1 (2.3)	
Features of MTM *, *n* (%) yes	Pre-APPE (*n* = 62)	Post-APPE (*n* = 44)	*p* value
MTM is patient centric	58 (93.5)	35 (79.5)	0.03
MTM is a comprehensive approach to improving medication use	57 (91.9)	40 (90.9)	0.85
MTM is product centric	3 (4.8)	5 (11.4)	0.21
MTM utilizes a CMR	57 (91.9)	40 (90.9)	0.85
MTM saves health insurance companies money	43 (69.4)	30 (68.2)	0.89
MTM allows for an interactive person-to-person, patient and provider conversation	58 (93.5)	36 (81.8)	0.06
MTM can be individualized for each patient	58 (93.5)	40 (90.9)	0.61
MTM can only be delivered by a pharmacist	12 (19.4)	9 (20.5)	0.89
None of the above	1 (1.6)	0 (0)	0.39

^+^ Pre APPE: 62 respondents provided 189 codes, average number of codes per respondent = 3.05. ^++^ Post APPE: 44 respondents provided 139 codes; average number of codes per respondent = 3.16. * could choose > 1 of the eight features. MRP = Medication related problem; CMR = Comprehensive Medication Review; HCP = Health Care Provider.

**Table 4:** Student Self-Confidence Engaging in MTM Service Delivery.

	Pre-APPE *n* = 62	Post-APPE *n* = 44	*p* Value
Overall Self-Confidence Delivering MTM (1 = Not at all Confident; 5 = Extremely Confident) Mean (SD)	3.27 (0.85)	4.02 (0.88)	<0.001
Agreement with specific MTM abilities (1 = Strongly Disagree; 5 = Strongly Agree), Mean (SD)			
Able to describe the features/benefits of MTM during recruitment	3.84 (0.85)	4.16 (1.08)	0.09
Able to describe the features/benefits of MTM to HCP or other stakeholders at site	3.81 (0.82)	4.02 (1.09)	0.25
Prepare for a CMR with a patient	3.68 (0.88)	4.32 (0.91)	<0.001
Complete a CMR session with a patient	3.76 (0.84)	4.23 (0.99)	0.01
Create a personal medication list for the patient	3.18 (1.0)	3.93 (1.26)	<0.001
Incorporate primary literature and reference sources to help resolve MRPs and transfer information	3.72 (0.83)	4.34 (0.81)	<0.001
Communicate potential MRPs verbally to the patient	3.94 (0.87)	4.5 (0.79)	<0.001
Communicate potential MRPs in writing to the patient via a Medication Action Plan	3.92 (0.84)	4.41 (0.76)	0.003
Prioritize and communicate in writing potential MRPs and/or recommendations to the HCP	3.81 (0.83)	4.36 (0.84)	0.001
Prioritize and verbally communicate MRPs and/or recommendations to the HCP	3.79 (0.79)	4.41 (0.94)	<0.001
Plan to practice MTM in future *n* (% Yes)	56 (90.3)	34 (77.3)	0.07

MTM = Medication Therapy Management, CMR = comprehensive medication review, MRP = Medication related problem, HCP = Health Care Provider.

**Table 5:** Student Self-Confidence Engaging in MTM Service Delivery post advanced pharmacy practice experiences (APPE), with and without self-reported APPE MTM learning opportunities.

	With APPE MTM Experience *N* = 30	Without APPE MTM Experience *N* = 14	*p* Value
	Mean (SD)	Mean (SD)	
Overall Self-Confidence Delivering MTM (1 = Not at all Confident; 5 = Extremely Confident) Mean (SD)	4.20 (0.71)	3.64 (1.08)	0.05
Agreement with specific MTM abilities (1 = Strongly Disagree; 5 = Strongly Agree), Mean (SD)			
Able to describe the features/benefits of MTM During recruitment	4.57 (0.63)	3.29 (1.33)	<0.001
Able to describe the features/benefits of MTM to HCP or other stakeholders at site	4.40 (0.81)	3.21 (1.19)	<0.001
Prepare for a CMR with a patient	4.57 (0.63)	3.79 (1.19)	0.01
Complete a CMR session with a patient	4.53 (0.78)	3.57 (1.09)	0.002
Create a personal medication list for the patient	4.23 (1.14)	3.29 (1.33)	0.02
Incorporate primary literature and reference sources to help resolve MRPs and transfer information	4.43 (0.73)	4.14 (0.95)	0.27
Communicate potential MRPs verbally to the patient	4.63 (0.61)	4.21 (1.05)	0.10
Communicate potential MRPs in writing to the patient via a Medication Action Plan	4.53 (0.68)	4.14 (0.86)	0.11
Prioritize and communicate in writing potential MRPs and/or recommendations to the HCP	4.50 (0.68)	4.07 (1.07)	0.12
Prioritize and verbally communicate MRPs and/or recommendations to the HCP	4.60 (0.62)	4.00 (1.36)	0.05
Plan to practice MTM in future *n* (%) Yes	25 (83.3)	9 (64.3)	0.17

## Supplementary Materials

The following are available online at http://www.mdpi.com/2226-4787/5/3/39/s1, Supplementary Formatsurvey tool, data files.

## References

[1] Centers for Medicare and Medicaid Services Medicare Part D Medication Therapy Management (MTM) Programs. CY 2014 Medication Therapy Management Program Guidance and Submission Instructions. https://www.cms.gov/medicare/prescription-drug-coverage/prescriptiondrugcovcontra/mtm.html.

[2] Viswanathan M., Kahwati L.C., Golin C.E., Blalock S., Coker-Schwimmer E., Posey R., Lohr K.N. Agency for Healthcare Research and Quality Medication Therapy Management Interventions in Outpatient Settings: A Systematic Review and Meta-analysis. http://effectivehealthcare.ahrq.gov/index.cfm/search-for-guides-reviews-and-reports/?productid=2000&pageaction=displayproduct.

[3] Bluml B.M. (2005). Definition of medication therapy management: Development of profession wide consensus. J. Am. Pharm. Assoc..

[4] American Pharmacists Association and the National Association of Chain Drug Stores Foundation (2008). Medication therapy management in pharmacy practice: Core elements of an MTM service model. J. Am. Pharm. Assoc..

[5] Patient-Centered Primary Care Collaborative The Patient-Centered Medical Home: Integrating Comprehensive Medication Management to Optimize Patient Outcomes June 2012. https://www.accp.com/docs/positions/misc/CMM%20Resource%20Guide.pdf.

[6] Joint Commission of Pharmacy Practitioners Pharmacists Patient Care Process. http://www.pharmacist.com/sites/default/files/files/PatientCareProcess.pdf.

[7] American Pharmacists Association Integrating Medication Therapy Management (MTM) Into the Curricula of Schools and Colleges of Pharmacy. http://www.pharmacist.com/sites/default/files/files/mtm_integrating_curricula_032012.pdf.

[8] Accreditation Council for Pharmacy Education Accreditation Standards and Key Elements for the Professional Program in Pharmacy Leading to the Doctor of Pharmacy degree. https://www.acpe-accredit.org/pdf/Standards2016FINAL.pdf.

[9] Medina M.S., Plaza C.M., Stowe C.D., Robinson E.T., DeLander G., Beck D.E., Melchert R.B., Supernaw R.B., Roche V.F., Gleason B.L. Center for the Advancement of Pharmacy Education (CAPE) Educational Outcomes 2013. http://www.aacp.org/resources/education/cape/Pages/default.aspx.

[10] Popovich N.G. (1991). Cultivating intellectual confidence in our students. Am. J. Pharm. Educ..

[11] Wongwiwatthananukit S., Newton G.D., Popovich N.G. (2002). Development and validation of an instrument to assess the self-confidence of students enrolled in the advanced pharmacy practice experience. Am. J. Pharm. Educ..

[12] Blake K.V., Madhavan S.S. (2010). Perceived barriers to provision of medication therapy management services (MTMS) and the likelihood of a pharmacist to work in a pharmacy that provides MTMS. Ann. Pharmacother..

[13] Martin B.A., Chui M.A., Thorpe J.M., Mott D., Kreling D.H. (2010). Development of a scale to measure pharmacists’ self-efficacy in performing medication therapy management services. Res. Soc. Adm. Pharm..

[14] Eukel H.N., Skoy E.T., Frenzel J.E. (2010). Provision of Medication Therapy Management to University Faculty and Staff Members by Third-year Pharmacy Students. Am. J. Pharm. Educ..

[15] Dahl J.R., Hall A.M. (2013). A scale to measure pharmacy students’ self-efficacy in performing Medication Therapy Management service. Am. J. Pharm. Educ..

[16] Standardized Framework for Cross-Walking Medication Therapy Management (MTM) Services to SNOWMED CT Codes. http://www.amcp.org/2016DraftSNOMEDProceedings/.

