# Supplementary material for: Experiential Education Builds Student Self-Confidence in Delivering Medication Therapy Management
Source: Pharmacy (Basel). 2017 Jul 11;5(3):39. doi: 10.3390/pharmacy5030039 (PMC5622351; doi:10.3390/pharmacy5030039)
Supplement: Supplementary file 1 [file pharmacy-05-00039-s001.zip › APPERotationStudy_W1.pdf]

APPE/Rotation Study

Thank you for agreeing to participate in this study today. We appreciate your time and feedback. Below are a series of questions that will help us learn about students in the Pharmacy Program. Please answer the questions to the best of your ability. Please note that this information will only be used in aggregate and none of the information will identify you as the respondent. The survey length will depend upon your answers and ranges from 5-10 minutes in length.

consent

Please indicate today's date to demonstrate your informed consent to complete this survey. ______________________ (MM-DD-YYYY)

Experience

Have you worked in a pharmacy in the past 2 years (not including experiential education/rotations)?

- ◯ Yes
- ◯ No

What type(s) of pharmacy have you worked in within the past 2 years (not including experiential education/rotations)? Please check all that apply.

- ☐ community chain (e.g., CVS, Walgreens)
- ☐ community independent
- ☐ community mass merchandiser (e.g., Target, Walmart)
- ☐ grocery store (e.g., Price Chopper, Wegmans)
- ☐ residency
- ☐ fellowship
- ☐ graduate school
- ☐ hospital
- ☐ long term care
- ☐ academia
- ☐ industry
- ☐ managed care
- ☐ other

Other work site

____

In the past 2 years, did you work in a pharmacy while classes were in session?

- ◯ Yes
- ◯ No

In the past 2 years, approximately how many hours in a typical week did you work in a pharmacy while classes were in session?

____
(Indicate your best estimate in the box above.)

In the past 2 years, did you work in a pharmacy during the summer or winter breaks?

- ◯ Yes
- ◯ No

In the past 2 years, approximately how many hours in a typical week did you work in a pharmacy over summer or winter break?

____
(Indicate your best estimate in the box above.)

After graduation, how likely are you to work in each of the following locations? For each site, please indicate your likelihood to work in that location on a scale of 1 to 5 where 5 is Extremely Likely and 1 is Not at all Likely.

	1=Not at all Likely	2	3	4	5=Extremely Likely
community chain	○	○	○	○	○
community independent	○	○	○	○	○
community mass merchandiser	○	○	○	○	○
grocery store	○	○	○	○	○
residency	○	○	○	○	○
fellowship	○	○	○	○	○
graduate school	○	○	○	○	○
hospital	○	○	○	○	○
long term care	○	○	○	○	○
academia	○	○	○	○	○
industry	○	○	○	○	○
managed care	○	○	○	○	○
other	○	○	○	○	○

After graduation which one site are you most likely to work in if you had to choose today?

○ community chain (e.g., CVS, Walgreens)
○ community independent
○ community mass merchandiser (e.g., Target, Walmart)
○ grocery store (e.g., Price Chopper, Wegmans)
○ residency
○ fellowship
○ graduate school
○ hospital
○ long term care
○ academia
○ industry
○ managed care
○ other
(Select only one answer.)

Other work after graduation ______________________________

What courses have you taken at ACPHS or elsewhere that you think were most helpful in preparing you for your APPE rotations? ______________________________

awareness

Do you know what Medication Therapy Management (MTM) services or programs are?

◯ Yes
◯ No
◯ Not Sure

In your own words, how would you define MTM?

____
(Please answer to the best of your ability.)

MTM part 2

From the list below, check the box next to the definitions that describe your understanding of MTM? You can select multiple definitions.

- ☐ MTM is patient centric
- ☐ MTM is a comprehensive approach to improving medication use
- ☐ MTM is product centric
- ☐ MTM utilizes a comprehensive medication review
- ☐ MTM saves health insurance companies money
- ☐ MTM allows for an interactive person-to-person patient and provider conversation
- ☐ MTM can be individualized for each patient
- ☐ MTM can only be delivered by a pharmacist
- ☐ None of these definitions

How did you first learn about MTM? __________________________________

Are you currently or in the future planning to complete an APPE in MTM or MTM-related services?

- ○ Yes
- ○ No

Did you complete the APhA MTM certificate training program?

- ○ Yes
- ○ No

Educational Evaluation: For the series of items below indicate your agreement with your abilities at this time, prior to your APPE rotations. For each question, please use the 1 to 5 scale where 1= strongly disagree and 5= strongly agree. We are looking for your fair assessment of your own skills for each of these items. We do not expect that you will be familiar with them all at this point.

	1=Strongly Disagree	2	3	4	5=Strongly Agree
I am able to describe the features and benefits of a MTM session when contacting patients to recruit them to participate in a MTM program.	○	○	○	○	○
I am able to describe the features and benefits of a MTM session to doctors or other stakeholders at the clinical site.	○	○	○	○	○
I can prepare for a Medication review session with a patient by conducting a preliminary review of prescription medication claims and diagnoses to identify potential medication related problems (MRP) (indication, safety, efficacy, compliance, etc).	○	○	○	○	○
I am able to complete a medication review session with a patient (either live or telephonically) including identifying the patient's chief compliant, conveying information in a patient appropriate manner, and using open questions.	○	○	○	○	○
I create a personal medication list (PML) for the patient using the web based MTM platform.	○	○	○	○	○
I can incorporate primary literature and print/electronic reference sources to create a plan to resolve identified MRPs and/or transfer of information among health practitioners, patients and the general public.	○	○	○	○	○

I can communicate the potential medication related problems (MRP or recommendations) to the patient verbally	○	○	○	○	○
I can communicate potential MRPs in writing to the patient via the Medication Action Plan above (patient friendly language) etc.	○	○	○	○	○
I can prioritize and communicate in writing the potential medication related problems (MRP) and/or recommendations to the physician via a written FARM/SOAP note using the web-based platform.	○	○	○	○	○
I can prioritize and verbally communicate potential medication related problems (MRP or recommendations) to the physician.	○	○	○	○	○

Are you interested or planning to offer MTM services following graduation wherever you will work?

- ○ Yes
- ○ No

How would you rate your self-confidence today to provide MTM services to patients? Please indicate your confidence on a scale of 1 to 5, where 5 is Extremely Confident and 1 is Not at all Confident?

- ○ 5=Extremely Confident
- ○ 4
- ○ 3
- ○ 2
- ○ 1=Not at all Confident

Will you be doing MTM related services this year on APPEs?

- ○ Yes
- ○ No
- ○ Not Sure

Which type of APPE do you expect to perform MTM related services?

- ○ Community Pharmacy
- ○ Inpatient Clinical
- ○ Institutional/Hospital
- ○ Ambulatory Care
- ○ Elective

Who is your preceptor for your APPE sites where you expect to be working on MTM services?

____

To which of the following APPE rotations have you been assigned for next year? Please check all that apply.

- ☐ MTM Elective - Dr. Cerulli
- ☐ Ambulatory Care Nephrology - Dr. Cardone
- ☐ Neither

demographics

What is your age?

- 17 and under
- 18-20
- 21-23
- 24-26
- 27 and older

Sex

- Male
- Female
- Transgender

On what ACPHS campus do you reside?

- Albany, NY
- Colchester, VT

What was your highest degree obtained prior to enrolling in the PharmD program?

- High School Diploma
- Associate's Degree
- Bachelor's Degree
- Master's Degree
- Doctoral Degree
- Other

What year will you or did you graduate from ACPHS?

- 2013
- 2014
- 2015
- 2016
- 2017
- 2018
- other

When do you plan to graduate? ________________________________

What is your current professional GPA?

- 3.5 or greater
- 3.0-3.49
- 2.5-2.99
- 2.0-2.49
- 1.9 or under

Incentive Drawing

Thank you for your participation. To thank you for your time and efforts, we would like to enter you in a raffle drawing for 1 of 5 $20 gift cards.

If you would like to participate in the drawing, please select yes and you will be asked for your contact information. Your email will not be associated with your survey responses and will only be used for the raffle drawing.

◯ Yes
◯ No

In order to be entered in the drawing, please enter your email address into the text box . If you do not want to participate in the raffle, you may skip this question.

____
